# Supplementary material for: Atomically dispersed Pb ionic sites in PbCdSe quantum dot gels enhance room-temperature NO2 sensing
Source: Nat Commun. 2021 Aug 12;12:4895. doi: 10.1038/s41467-021-25192-4 (PMC8361172; doi:10.1038/s41467-021-25192-4)
Supplement: Supplementary file 1 — Supplementary Information [file 41467_2021_25192_MOESM1_ESM.pdf]

Supplementary Information

Atomically dispersed Pb ionic sites in PbCdSe quantum dot gels enhance room-temperature NO_2 sensing

Xin Geng,[1,‡] Shuwei Li,[2, 3, 4,‡] Lalani Mawella-Vithanage,[1] Tao Ma,[5] Mohamed Kilani,[6] Bingwen Wang,[7] Lu Ma,[8] Chathuranga C. Hewa-Rahinduwage,[1] Alina Shafikova,[1] Eranda Nikolla,[7] Guangzhao Mao,[6] Stephanie L. Brock,[1,*] Liang Zhang,[2, 3, 4, *] and Long Luo[1,*]

[1] Department of Chemistry, Wayne State University, Detroit, Michigan 48202, United States

[2] Center for Combustion Energy, Tsinghua University, Beijing 100084, China

[3] School of Vehicle and Mobility, Tsinghua University, Beijing 100084, China

[4] State Key Laboratory of Automotive Safety and Energy, Beijing 100084, China

[5] Michigan Center for Materials Characterization, University of Michigan, Ann Arbor, Michigan 48109, United States

[6] School of Chemical Engineering, University of New South Wales, Sydney, NSW 2052, Australia

[7] Department of Chemical Engineering and Material Science, Wayne State University, Detroit, Michigan 48202, United States

[8] National Synchrotron Light Source II, Brookhaven National Laboratory, Upton, NY 11973

Corresponding Authors:

*sbrock@chem.wayne.edu

*zhangbright@tsinghua.edu.cn

*long.luo@wayne.edu

Contents

I. Supplementary Tables

Supplementary Table 1. Elemental composition of $Pb_xCd_{1-x}Se$ gels.

Supplementary Table 2. BET data of various gels.

Supplementary Table 3. XPS peak analysis of Pb4$f_{5/2}$ region for $Pb_xCd_{1-x}Se$ QD gels with x=0.02, 0.04, 0.09, 0.17, 0.40 and 1.0.

Supplementary Table 4. Structural parameters obtained by fitting the Cd K-edge EXAFS data.

Supplementary Table 5. Comparison of sensing performance for $Pb_{0.09}Cd_{0.91}Se$ gel sensors with different thickness towards 1.32 ppm NO_2 at room temperature.

Supplementary Table 6. Sensor response (S), response time (t_{res}), and recovery time (t_{rec}) of $Pb_xCd_{1-x}Se$ QD gels towards 1.32 ppm NO_2 as a function of x.

Supplementary Table 7. Comparison of the state-of-arts room-temperature NO_2 gas sensors based on p-type materials.

II. Supplementary Figures

Supplementary Figure 1. Size distributions of **a**, CdSe QDs; **b**, CdSe gel; **c**, $Pb_{0.04}Cd_{0.96}Se$ gel; **d**, $Pb_{0.09}Cd_{0.91}Se$ gel; **e**, $Pb_{0.17}Cd_{0.83}Se$ gel; **f**, $Pb_{0.40}Cd_{0.60}Se$ gel; and **g**, PbSe gel.

Supplementary Figure 2. The photographs of $Pb_xCd_{1-x}Se$ wet gels (x=0, 0.003, 0.02, 0.04, 0.09, 0.17, 0.40 and 1.0).

Supplementary Figure 3. a, Low and **b**, high-magnification TEM images of PbSe QD gel.

Supplementary Figure 4. Photographs of $Pb_xCd_{1-x}Se$ aerogels (x=0, 0.09, 0.17 and 0.40).

Supplementary Figure 5. a, Low and **b**, high-magnification HAADF-STEM images of $Pb_{0.04}Cd_{0.96}Se$ gel. **c**, Low and **d**, high-magnification HAADF-STEM images of $Pb_{0.17}Cd_{0.83}Se$ gel. **e**, Low and **f**, high-magnification HAADF-STEM images of $Pb_{0.40}Cd_{0.60}Se$ gel.

Supplementary Figure 6. Integrated EDS elemental mappings of **a**, $Pb_{0.04}Cd_{0.96}Se$; **b**, $Pb_{0.09}Cd_{0.91}Se$; **c**, $Pb_{0.17}Cd_{0.83}Se$; **d**, $Pb_{0.40}Cd_{0.60}Se$ QD gels.

Supplementary Figure 7. XPS peak analysis of Pb4$f_{5/2}$ region for $Pb_xCd_{1-x}Se$ QD gels with x=0.02, 0.04, 0.09, 0.17, 0.40 and 1.0.

Supplementary Figure 8. a, XANES spectra of CdSe, $Pb_{0.09}Cd_{0.91}Se$, and $Pb_{0.4}Cd_{0.6}Se$ QD gels at Cd K-edge. The EXAFS spectra and fitting results of **b**, CdSe gel; **c**, $Pb_{0.09}Cd_{0.91}Se$ gel; **d**, $Pb_{0.4}Cd_{0.6}Se$ gel.

Supplementary Figure 9. XANES spectra of PbSe, $Pb_{0.09}Cd_{0.91}Se$, and $Pb_{0.4}Cd_{0.6}Se$ QD gels at **a**, Pb L_{III} edge; **b**, Pb L_{II} edge and **c**, Pb L_I edge. The corresponding expanded views at **d**, Pb L_{III} edge; **e**, Pb L_{II} edge; and **f**, Pb L_I edge.

Supplementary Figure 10. Flowchart for the active machine learning process.

Supplementary Figure 11. The searching process of the most stable geometries at each composition in the last run of the Monte Carlo simulation.

Supplementary Figure 12. The relative formation energy per formular unit for cubic phase $Pb_xCd_{1-x}Se$.

Supplementary Figure 13. The change of MAE and R^2 as a function of the number of active learning loops.

Supplementary Figure 14. The parity plot of cubic and hexagonal phase in the last iteration.

Supplementary Figure 15. The most stable structures for each composition searched by our scheme.

Supplementary Figure 16. Schematic of the sensor substrate fabrication.

Supplementary Figure 17. Geometry and dimensions of the sensor electrodes.

Supplementary Figure 18. **a**, Low and **b**, high-magnification Field Emission Scanning Electron Microscope (FE-SEM) surface morphology images of $Pb_{0.09}Cd_{0.91}Se$ gel sensor. **c**, TEM image of $Pb_{0.09}Cd_{0.91}Se$ gel sensor film.

Supplementary Figure 19. FE-SEM cross-sectional images of $Pb_{0.09}Cd_{0.91}Se$ gel sensors prepared by drop-casting **a**, 2 μL; **b**, 10 μL; and **c**, 50 μL wet gel onto the sensor substrate, respectively. **d**, Response-recovery curves of $Pb_{0.09}Cd_{0.91}Se$ gel sensors with a thickness of 0.7 μm, 3.1 μm, and 18.5 μm at 1.32 ppm NO_2 at room temperature.

Supplementary Figure 20. **a**, Nitrogen adsorption-desorption isotherms; **b**, Barrett–Joyner–Halenda pore size distributions of $Pb_{0.09}Cd_{0.91}Se$ xerogel and A-xerogel. **c**, Cross-section FE-SEM image of $Pb_{0.09}Cd_{0.91}Se$ A-xerogel sensor; **d**, FE-SEM and **e**, TEM image of the $Pb_{0.09}Cd_{0.91}Se$ A-xerogel sensor film; **f**, Response-recovery curve of $Pb_{0.09}Cd_{0.91}Se$ xerogel and A-xerogel sensors in response to 1.32 ppm NO_2 at room temperature.

Supplementary Figure 21. **a**, Schematic diagram of measuring contact resistance. **b**, Extrapolated line fitting for determining the contact resistance from total resistance versus distance between electrodes relationship of $Pb_{0.09}Cd_{0.91}Se$ gel.

Supplementary Figure 22. Limit of detection (LOD) of $Pb_xCd_{1-x}Se$ gel sensors (x=0, 0.003, 0.02, 0.04, 0.09, 0.17, 0.40 and 1.0).

Supplementary Figure 23. **a**, XRD patterns of $Pb(NO_3)_2$@CdSe gel, CdSe gel and $Pb(NO_3)_2$. **b**, XPS results of the Pb4$f_{5/2}$ region for $Pb(NO_3)_2$@CdSe gel and $Pb(NO_3)_2$. **c**, Response–recovery curve of $Pb(NO_3)_2$@CdSe gel sensor to NO_2 (3 ppb–1.32 ppm) at room temperature.

Supplementary Figure 24. A typical electrical resistance-time trace for a $Pb_{0.09}Cd_{0.91}Se$ gel sensor during the response-recovery test.

Supplementary Figure 25. Photograph of the home-made wireless portable device, and the schematic of the voltage divider of the portable device.

Supplementary Figure 26. Photographs of the readouts of a $Pb_{0.09}Cd_{0.91}Se$ gel portable device and a commercial NO_2 device purchased from amazon (Purchase link: https://www.amazon.com/FORENSICS-Anti-slip-Explosion-Adjustable-Vibration/dp/B07BDNYR86) at different NO_2 concentrations of **a**, 600 ppb; **b**, 300 ppb; **c**, 200 ppb; **d**, 100 ppb; **e**, 80 ppb; **f**, 60 ppb; **g**, 50 ppb; **h**, 30 ppb; and **i**, 10 ppb.

Supplementary Figure 27. The optimal adsorption geometries (top and side view) for NO_2 adsorption on various surface structures.

Supplementary Figure 28. The most stable adsorption geometries (top and side view) for various interfering gases adsorbed on D2.

III. Supplementary Movie 1

I.Supplementary Tables

Supplementary Table 1. Elemental composition of $Pb_xCd_{1-x}Se$ gels analyzed by X-ray Photoelectron Spectroscopy (XPS) and Inductively Coupled Plasma Mass Spectrometry (ICP-MS).

	[Pb]/([Pb]+[Cd])			[Se]/([Pb]+[Cd])
Samples	XPS	ICP-MS	Avg.	XPS
0	0	0	0	1.12
3 mM	0.003	0.003	0.003	1.12
6 mM	0.022	0.019	0.02	1.12
12.5 mM	0.044	0.040	0.04	1.11
25 mM	0.087	0.092	0.09	1.11
50 mM	0.172	0.168	0.17	1.12
150 mM	0.402	0.399	0.40	1.11
750 mM	1	1	1	1.11

Supplementary Table 2. BET data of CdSe aerogel, $Pb_{0.09}Cd_{0.91}Se$ aerogel, $Pb_{0.17}Cd_{0.83}Se$ aerogel, $Pb_{0.40}Cd_{0.60}Se$ aerogel, $Pb_{0.09}Cd_{0.91}Se$ xerogel and A-xerogel. Xerogel was prepared by drying $Pb_{0.09}Cd_{0.91}Se$ wet gel under ambient conditions, while A-xerogel was prepared by mixing aerogel with methanol to make a slurry and then drying it under ambient conditions.

Sample	BET surface area (m^2/g)	BJH average pore diameter (nm)	BJH cumulative pore volume (cm^3/g)
CdSe aerogel	209.4	27.4 (adsorption isotherm)	1.2 (adsorption isotherm)
		22.3 (desorption isotherm)	1.3 (desorption isotherm)
$Pb_{0.09}Cd_{0.91}Se$ aerogel	205.2	31.2 (adsorption isotherm)	1.5 (adsorption isotherm)
		22.5 (desorption isotherm)	1.5 (desorption isotherm)
$Pb_{0.17}Cd_{0.83}Se$ aerogel	199.8	34.5 (adsorption isotherm)	1.4 (adsorption isotherm)
		25.4 (desorption isotherm)	1.4 (desorption isotherm)
$Pb_{0.40}Cd_{0.60}Se$ aerogel	177.0	21.3 (adsorption isotherm)	0.8 (adsorption isotherm)
		17.9 (desorption isotherm)	0.8 (desorption isotherm)
$Pb_{0.09}Cd_{0.91}Se$ xerogel	30.4	3.0 (adsorption isotherm)	0.2 (adsorption isotherm)
		2.9 (desorption isotherm)	0.3 (desorption isotherm)
$Pb_{0.09}Cd_{0.91}Se$ A-xerogel	35.1	6.4 (adsorption isotherm)	0.1 (adsorption isotherm)
		5.4 (desorption isotherm)	0.1 (desorption isotherm)

Supplementary Table 3. XPS peak analysis of Pb4$f_{5/2}$ region for $Pb_xCd_{1-x}Se$ QD gels with x=0.02, 0.04, 0.09, 0.17, 0.40 and 1.0. Fitting was performed using a composite function (30% Lorentzian + 70% Gaussian).

		Atomically dispersed sites	Cubic phase	Atomically dispersed sites (%)	Cubic phase (%)
$Pb_{0.02}Cd_{0.98}Se$	Binding energy (eV)	143.47	143.82	100	0
	Area ratio	1	0		
$Pb_{0.04}Cd_{0.96}Se$	Binding energy (eV)	143.52	143.82	100	0
	Area ratio	1	0		
$Pb_{0.09}Cd_{0.91}Se$	Binding energy (eV)	143.50	143.81	100	0
	Area ratio	1	0		
$Pb_{0.17}Cd_{0.83}Se$	Binding energy (eV)	143.46	143.84	44.4	55.6
	Area ratio	0.80	1		
$Pb_{0.40}Cd_{0.60}Se$	Binding energy (eV)	143.45	143.78	16.0	84.0
	Area ratio	0.19	1		
PbSe	Binding energy (eV)	143.49	143.77	0	100
	Area ratio	0	1		

Supplementary Table 4. Structural parameters obtained by fitting the Cd K-edge EXAFS data. CN, coordination number; R, distance between absorber and backscatter atoms; ΔR, the uncertainty of R; σ^2, Debye–Waller factor to account for both thermal and structural disorders; R factor, indicates the goodness of the fit.

Sample	Path	CN	R (Å)	ΔR (Å)	σ^2 (10^{-3} Å)	R-factor
CdSe	Cd-Se	3.4	2.606	0.083	0.00503	0.0141
	Cd-Cd	10.0	3.732	0.117	0.0381	0.0233
$Pb_{0.09}Cd_{0.91}Se$	Cd-Se	2.8	2.571	0.075	0.00506	0.0124
	Cd-Cd	8.5	3.844	-0.337	0.02847	0.0258
$Pb_{0.4}Cd_{0.6}Se$	Cd-Se	3.8	2.602	0.0875	0.00547	0.0170
	Cd-Cd	11.7	3.937	0.256	0.02914	0.027

Supplementary Table 5. Comparison of sensing performance for $Pb_{0.09}Cd_{0.91}Se$ gel sensors with different thicknesses towards 1.32 ppm NO_2 at room temperature.

	Sensor response (S, %)	Response time (t_{res}, s)	Recovery time (t_{rec}, s)	Base resistance (MΩ)
0.7 μm	75.5	26	61	31.7
3.1 μm	72.8	28	66	5.8
18.5 μm	66.7	35	82	1.0

Supplementary Table 6. Sensor response (S), response time (t_{res}), and recovery time (t_{rec}) of $Pb_xCd_{1-x}Se$ QD gels towards 1.32 ppm NO_2 as a function of x.

x in $Pb_xCd_{1-x}Se$	Sensor response (S, %)		Response time (t_{res}, s)	Recovery time (t_{rec}, s)
0	5.2		24	31
0.003	6.8		25	34
0.02	44.7	1300%	26	46
0.04	52.4		27	53
0.09	72.8		28	66
0.17	83.5	1786%	29	85
0.4	93.7	486%	30	150
1	98.1		30	240

Supplementary Table 7. Comparison of the state-of-arts room-temperature NO_2 gas sensors based on p-type materials.

Materials	Methods	S, %/ppb	t_{res}/t_{rec} (s)	LOD (ppb)	References
Cu_2O nanowires/reduced graphene oxide	Hydrothermal	0.033	380/590	400	*J. Am. Chem. Soc.* **134**, 4905-4917 (2012)
Cu_2O/CuO mesocrystal nanoflower	Etch-oriented self-assembly	0.0075	60/110	100	*J. Mater. Chem. A,* **6**, 17120-17131 (2018)
CuO/graphene nanosheets	Precipitation	0.0009	58/200	97	*Nanoscale* **6**, 7369-7378 (2014)
CuO nanoplatelets	Sonochemical	0.0001	150/180	5000	*Sens. Actuators B* **266**, 761-772 (2018)
CuO/reduced graphene oxide nanohybrids	One-pot solution process	0.014	110/200	1000	*Sens. Actuators B* **271**, 306-310 (2018)
CuO nanoplatelets	Wet chemical	0.0225	270/300	10000	*ACS Omega* **4**, 18035-18048 (2019)
CuO/carbon nanotube nanohybrids	Reflux	0.0001	130/170	970	*Appl. Surf. Sci.* **428**, 415-421 (2018)
CuO/NiO nanohybrids	Reflux and hydrothermal	0.0008	100/200	1000	*Appl. Surf. Sci.* **412**, 230-237 (2017)
CuO/porous silicon film	Sputtering	0.068	51/547	125	*J. Alloys Compd.* **685**, 364-369 (2016)
CuO/ZnO film	High-temperature oxidation	0.0022	200/1100	1000	*R. Soc. open sci.* **5**, 171788 (2018)
Zn-doped CuO/ reduced graphene oxide nanohybrids	Wet chemical	0.0011	300/2000	6000	*J. Mater. Sci. Mater. Electron.* **29**, 10640-10655 (2018)
CuO/carbon fibers	Electroless plating	0.0001	140/2400	100000	*J. Ind. Eng. Chem.* **60**, 341-347 (2018)
NiO/reduced graphene oxide nanosheets	Hydrothermal	0.007	576/121	250	*Phys. Chem. Chem. Phys.* **17**, 14903-14911 (2015)
Al doped NiO nanosheets	Microwave assisted solvent-thermal	0.022	95/100	250	*Phys. Chem. Chem. Phys.* **19**, 19043-19049 (2017)

NiO/WO_3 nanoplates	Hydrothermal	0.01	125/111	5000	*Nanoscale* **6**, 4063-4066 (2014)
NiO nanosheets	Hydrothermal	0.004	100/300	7000	*J. Phys. Chem. C* **119**, 17930−17939 (2015)
NiO mesoporous nanosheets	Hydrothermal	0.005	30/200	5000	*J. Phys. Chem. C* **120**, 3936-3945 (2016)
NiO:Ce nanoparticles	Sol-gel	0.075	60/1000	10000	*J. Phys. Chem. Solids,* **114**, 28-35 (2018)
NiO nanosheets/TiO_2 QDs	Wet chemical	0.03	700/1000	5000	*Nanomaterials* **9**, 1628 (2019)
NiO/SnO_2/reduced graphene oxide	Ball milling	0.05	220/835	5000	*Sens. Actuators B* **243**, 1010-1019 (2017)
NiO thin film	Sputtering	0.026	300/750	1000	*Thin Solid Films* **418**, 9-15 (2002)
NiO/reduced graphene oxide nanohybrids	Wet chemical	0.0043	49/62	3000	*Mater. Res. Bull.* **84**, 168-176 (2016)
NiO nanosheets	Hydrothermal	0.035	792/1746	57	*Ceram. Int.* **45**, 4253-4261 (2019)
NiO thin film	Sol-gel	0.013	60/180	1000	*Ceram. Int.,* **44**, 753-759 (2018)
NiO/polypyrrole nanohybrids	Spin-coating	0.0005	100/1200	10000	*Ionics* **20**, 1607-1616 (2014)
2D NiO nanosheet/2D graphene nanohybrids	Wet chemical	0.02	150/270	1000	*Sens. Actuators B* **185**, 701-705 (2013)
Porous Co_3O_4 slices/ reduced graphene oxide nanohybrids	Hydrothermal	0.006	90/2400	50	*Sens. Actuators B* **263**, 387-399 (2018)
Co_3O_4/reduced graphene oxide nanohybrids	Hydrothermal	0.001	600/3600	60000	*Sens. Actuators B* **188**, 902-908 (2013)
Co_3O_4/Al-ZnO nanohybrids	Wet chemical	0.04	120/600	12500	*Sensors* **19**, 760 (2019)
Co_3O_4/TiO_2 nanotubes	Electrochemical deposition	0.0003	250/300	5000	*Sensors* **18**, 956 (2018)
Mn_3O_4 nanorods	Hydrothermal	0.009	116/100	5000	*Sens. Actuators B* **285**, 92-107 (2019)
Te thin film	Vacuum thermal evaporation	0.06	180/900	80	*Sens. Actuators B* **73**, 35-39 (2001)
Te nanofibers	Electrospinning	0.006	84/780	100	*Nanoscale* **5**, 3058-3062 (2013)
WS_2 nanosheets/Ag nanowires	Atomic layer deposition	0.001	180/300	25000	*ACS Nano* **10**, 9287-9296 (2016)
WS_2 nanosheets /multiple carbon nanofibers	Electrospinning	0.017	860/890	1000	*J. Mater. Chem. A* **5**, 8725-8732 (2017)
WS_2 aerogel	Wet chemical	0.0001	850/900	2000	*FlatChem* **5**, 1-8 (2017)
WS_2/indium-gallium-zinc-oxide (IGZO) film	Chemical vapor deposition	0.01	1200/1800	1000	*ACS Appl. Mater. Interfaces* **11**, 40850−40859 (2019)
MoS_2 nanosheets	Mechanically exfoliation	0.0013	300/600	100000	*ACS Nano* **7**, 4879-4891 (2013)
MoS_2 nanosheets	Chemical vapor deposition	0.08	870/895	20	*ACS Nano* **8**, 5304-5314 (2014)

MoS_2 nanosheets	Chemical vapor deposition	0.09	60/1800	120	*ACS Appl. Mater. Interfaces* **7**, 2952-2959 (2015)
MoS_2 thin films	Chemical vapor deposition	0.0023	60/600	1500	*Sci. Reports* **5**, 8052 (2015)
$MoSe_2$ films	Chemical vapor deposition	0.006	120/120	60000	*Nano Res.* **10**, 1861-1871 (2017)
Au/MoS_2 nanosheets	Chemical vapor deposition	0.049	600/500	25	*ACS Nano* **13**, 3196-3205 (2019)
Pt/MoS_2 nanosheets	Exfoliation	0.01	1800/3600	500	*Small* **8**, 2994-2999 (2012)
MoS_2/SnO_2 nanosheets	Thermal oxidation	0.0028	408/162	500	*Small* **11**, 2305-2313 (2015)
PbS colloidal quantum dots	Hot injection/spin coating	0.05	22/57	84	*Adv. Mater.* **26**, 2718-2724 (2014)
PbS/ZnO nanohybrids	Precipitation	0.012	180/240	1000	*Sens. Actuators B* **255**, 2538-2545 (2018)
CdS/ZnO film	Liquid plasma spray	0.031	1000/2400	1000	*Ceram. Int.* **42**, 4845-4852 (2016)
SnS_2/reduced graphene oxide nanohybrids	Hydrothermal	0.047	90/150	125	*J. Mater. Chem. C* **7**, 8616-8625 (2019)
$MoTe_2$ nanosheets	Mechanical exfoliation	0.015	240/250	20	*ACS Sens.* **3**, 1719-1726 (2018)
p-type SnO_x thin film	Sputtering	0.002	150/1200	1000	*Sens. Actuators B* **288**, 625-633 (2019)
$CuInS_2$ QDs decorated ring-like NiO	Wet chemical	0.005	300/500	10000	*Sens. Actuators B* **256**, 1001-1010 (2018)
Graphene film	Spin coating	0.0054	600/900	5000	*ACS Nano* **3**, 301-306 (2009)
Graphene film	Epitaxial	0.02	300/1500	262	*ACS Sens.* **3**, 1666-1674 (2018)
Graphene film	Epitaxial	0.004	210/240	500	*Sens. Actuators B* **150**, 301-307 (2010)
Graphene film	Epitaxial	0.07	100/1000	20	*Sens. Actuators B* **236**, 1054-1060 (2016)
Graphene film	Chemical vapor deposition/gating	0.0014	90/300	20000	*Appl. Phys. Lett.* **102**, 043101 (2013)
Graphene film	Chemical vapor deposition	0.0001	120/150	1000	*ACS Omega* **4**, 14179-14187 (2019)
Graphene film	Epitaxial	0.01	2700/3000	200	*J. Sens.* **2015**, 7 (2015)
Graphene film	Chemical vapor deposition	0.052	150/1200	200	*RSC Adv.* **6**, 84082-84089 (2016)
Graphene film	Chemical vapor deposition	0.025	1000/1200	100	*Appl. Phys. Lett.* **100**, 203120 (2012)
Single layered graphene	Epitaxially grown	0.0004	3000/10000	2500	*Sens. Actuators B* **155**, 451-455 (2011)
Ozone treated graphene	Chemical vapor deposition	0.001	900/1800	200	*Sens. Actuators B* **166-167,** 172-176 (2012)
Graphene nanomesh	Chemical vapor deposition/ nanomesh	0.006	1000/1200	1000	*Anal. Chem.,* **84**, 8171-8178 (2012)
Graphene nanosheets	Thermal reduction	0.0015	600/1800	2000	*Nanotechnology*, **20**, 445502 (2009)

Graphene nanosheets	Thermal reduction	0.003	1000/2000	1000	*Carbon* **91**, 178-187 (2015)
N and Si co-doped graphene nanosheets	High-temperature annealing	0.0013	68/635	1000	*J. Mater. Chem. A* **1**, 6130-6133 (2013)
Caesium doped graphene oxide	Thermal solid-state reaction	0.003	245/2200	91	*Beilstein J. Nanotechnol.* **5**, 1073-1081 (2014)
Reduced graphene oxide film	Chemical vapor deposition	0.0013	800/1300	1000	*Sens. Actuators B* **190**, 865-872 (2014)
Reduced graphene oxide film	Two-beam-laser interference	0.0015	34/45	4000	*Sci. Rep.* **8**, 4918 (2018)
Reduced graphene oxide film	Chemical reduction	0.05	250/500	50	*Anal. Chem.* **86**, 7516-7522 (2014)
Reduced graphene oxide/Pd nanosheets	Chemical vapor deposition	0.05	71/5000	22	*ACS Nano* **5**, 6955-6961 (2011)
Reduced graphene oxide/poly(3-hexylthiophene) film	Wet chemical	0.0062	300/1800	2000	*Chem. Phys. Lett.* **614**, 275-281 (2014)
Reduced graphene oxide/Ag nanowire	Dip-coating	0.0003	350/850	5000	*Sens. Actuators B* **265**, 609-616 (2018)
Reduced graphene oxide/Cu-modified carbon spheres	Hydrothermal	0.0017	84/370	10000	*Chem. Phys. Lett.* **695**, 153-157 (2018)
Reduced graphene oxide nanofibrous mesh fabric	Electrospinning	0.014	1000/5000	1000	*Sens. Actuators B* **257**, 846-852 (2018)
Sulfonated reduced graphene oxide/Ag nanoparticles	Gravure printing	0.0014	120/200	500	*ACS Appl. Mater. Interfaces* **6**, 7426-7433 (2014)
Reduced graphene oxide/ZnO nanowalls	Thermal reduction and soft solution	0.05	50/58	5000	*Appl. Surf. Sci.* **423**, 721-727 (2017)
Graphene aerogel/ZnO nanospheres	Solvothermal	0.0001	149/243	10000	*Sens. Actuators B* **211**, 220-226 (2015)
Reduced graphene oxide/hydrazine/ZnO nanoparticles	Wet chemical	0.005	165/499	1000	*Sens. Actuators B* **202**, 272-278 (2014)
Reduced graphene oxide/ZnO nanoparticles	Hydrolysis	0.0002	161/1552	5000	*Eur. J. Inorg. Chem.* **2015**, 1912-1923 (2015)
Reduced graphene oxide/ZnO nanorods	Anchored seeding/ oriented growth	0.055	120/240	1000	*ACS Appl. Mater. Interfaces* **8**, 35454-35463 (2016)
Reduced graphene oxide/ZnO nanoparticles	Reflux	0.0012	100/2400	4000	*AIP Conference Proceedings* **1953**, 030039 (2018)
Graphene-wrapped WO_3 nanosheets	Sol-gel	0.002	200/250	7000	*Sens. Actuators B* **220**, 201-209 (2015)
Reduced graphene oxide/ WO_3 nanosheets	Metal organic decomposition	0.031	600/7200	500	*Talanta* **132**, 398-405 (2015)
Reduced graphene oxide/Fe-doped WO_3 nanoparticles	Sputtering	0.0019	1500/7200	1000	*Appl. Surf. Sci.* **434**, 126-133 (2018)
Reduced graphene oxide/In_2O_3 nanosheets	Hydrothermal	0.027	240/1440	5000	*Sens. Actuators B* **219**, 94-99 (2015)

Reduced graphene oxide/In_2O_3 nanoflowers	Hydrothermal	0.024	240/1440	10	*J. Colloid Interface Sci.* **504**, 206-213 (2017)
Graphene oxide/In_2O_3 cubes	Microwave assisted hydrothermal	0.012	180/240	1000	*ACS Appl. Mater. Interfaces* **6**, 21093-21100 (2014)
Reduced graphene oxide/ In_2O_3 nanoparticles	Modified Hummers	0.031	252/798	50	*Sens. Actuators B* **277**, 114-120 (2018)
Reduced graphene oxide/ $In(OH)_3$ nanoparticles	Hydrothermal	0.016	160/180	1000	*Sens. Actuators B* **214**, 36-42 (2015)
Carbon nanotubes	Inkjet printing	0.005	180/600	64	*J. Am. Chem. Soc.*, **134**, 4553-4556 (2012)
Carbon nanotubes	Thermal evaporation	0.0005	570/960	44	*Nano Lett.* **3**, 929-933 (2003)
Au nanowires/carbon nanotubes	Thermal evaporation	0.01	870/3600	100	*ACS Nano* **5**, 4592-4599 (2011)
Polyethyleneimine/ carbon nanotubes	Chemical vapor deposition	0.05	90/120	10	*Nano Lett.* **3**, 347-351 (2003)
Multi-walled carbon nanotubes/polyaniline	Chemical vapor deposition	0.0003	880/3600	100000	*Nanotechnology* **22**, 215502 (2011)
Multi-walled carbon nanotubes/WO_3 nanoparticles	Metal organic decomposition	0.002	800/1000	100	*Mater. Chem. Phys.* **125**, 351-357 (2011)
Multi-walled carbon nanotubes/WO_3 nanoparticles	Hydrothermal	0.0028	590/1620	1000	*Sens. Actuators B* **221**, 760-768 (2015)
Polypyrrole/WO_3 thin film	Sol-gel	0.0006	900/10000	5000	*Org. Electron.* **16**, 195-204 (2015)
Polypyrrole/ 4-dodecyl-benzenesulfonic /WO_3 nanohybrids	Solid state synthesis	0.0007	300/5700	5000	*Org. Electron.* **19**, 15-25 (2015)
Organic heterojunction	Thermal evaporation	0.026	1800/3600	5000	*Adv. Mater.*, **25**,1755-1760 (2013)
Phosphorene nanosheets	Exfoliation	0.016	600/1200	20	*Nat. Comm.* **6**, 8632 (2015)
Polyaniline fibers	Electrospinning	0.08	50/70	50	*Adv. Funct. Mater.* **24**, 4005-4014 (2014)
Polythiophene film	Chemical oxidative polymerization	0.0009	297/400	10000	*Synth. Met.* **195**, 228-233 (2014)
Polystyrene bead decorated graphene	Chemical vapor deposition	0.005	300/1200	250	*ACS Omega* **4**, 3812-3819 (2019)
Polypyrrole/camphor sulfonic acid doped ZnO	Spin coating	0.0015	290/3600	10000	*Synth. Met.* **162**, 1598-1603 (2012)
Poly(3-hexylthiophene) /ZnO nanowires	Vapor deposition	0.008	295/120	4000	*Appl. Phys. Lett.* **90**, 043516 (2007)
Polypyrrole/ZnO thin film	Chemical polymerization/ sol-gel	0.0004	240/8640	10000	*Measurement* **45**, 1986-1996 (2012)
Poly(3-hexylthiophene) /ZnO thin film	Spin coating	0.0036	900/2700	1000	*ACS Appl. Mater. Interfaces* **8**, 8600-8607 (2016)

Porous silicon/ZnO nanorods	Hydrothermal	0.0007	1000/1500	5000	*Phys. Chem. Chem. Phys.* **18**, 4835-4841 (2016)
CdS QDs gel	Electrogelation	0.009	29/28	11	*J. Am. Chem. Soc.* **142**, 12207–12215 (2020)
CdSe QDs gel	Electrogelation	0.004	24/31	440	This work
PbS QDs gel	Electrogelation/ Complete cation exchange	0.08	33/300	3	This work
PbSe QDs gel	Electrogelation/ Complete cation exchange	0.075	30/240	3	This work
$Pb_{0.09}Cd_{0.91}Se$ gel	Electrogelation/ Partial cation exchange	0.06	28/60	3	This work

II. Supplementary Figures

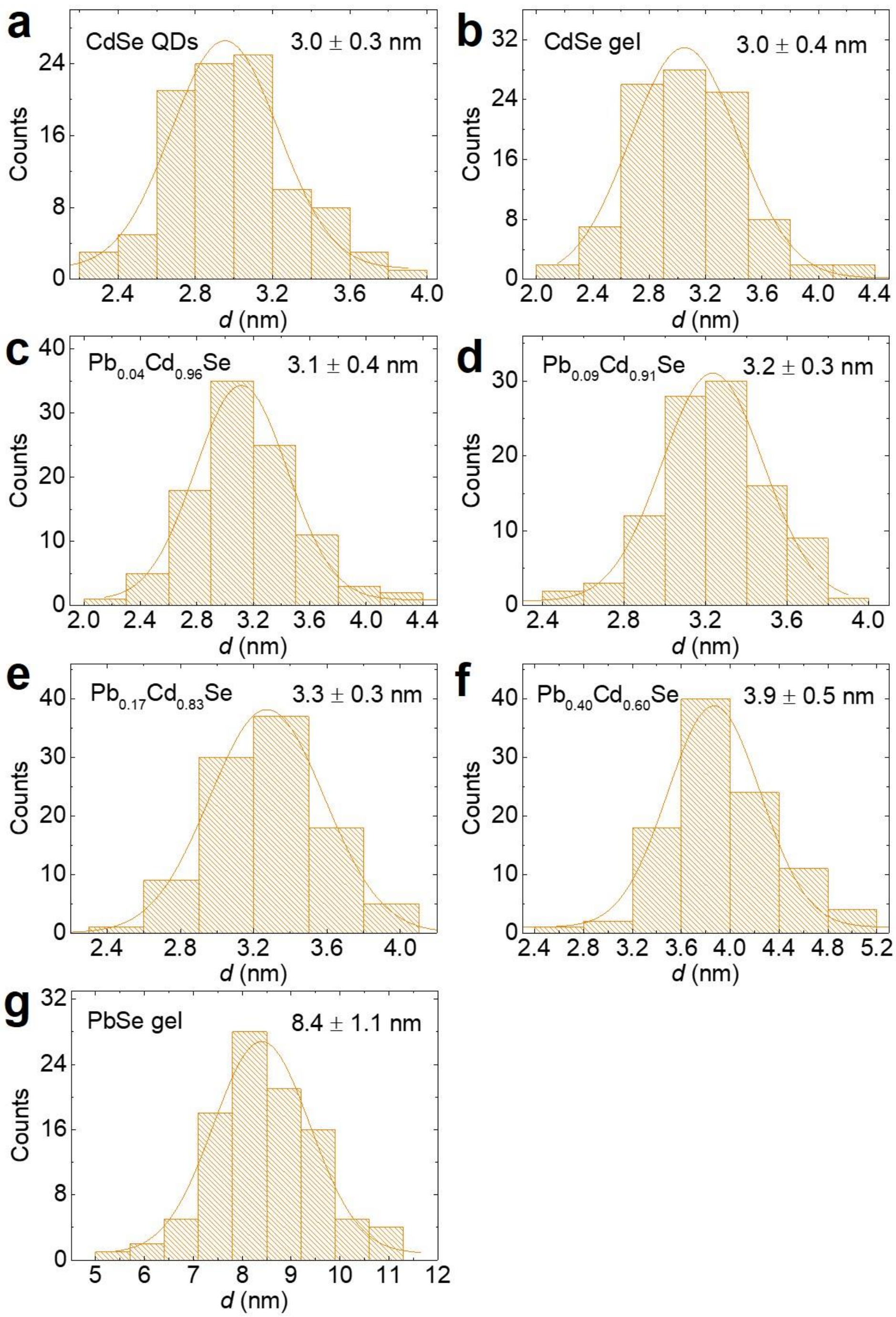

Supplementary Figure 1. Size distributions of **a**, CdSe QDs; **b**, CdSe gel; **c**, $Pb_{0.04}Cd_{0.96}Se$ gel; **d**, $Pb_{0.09}Cd_{0.91}Se$ gel; **e**, $Pb_{0.17}Cd_{0.83}Se$ gel; **f**, $Pb_{0.40}Cd_{0.60}Se$ gel; and **g**, PbSe gel.

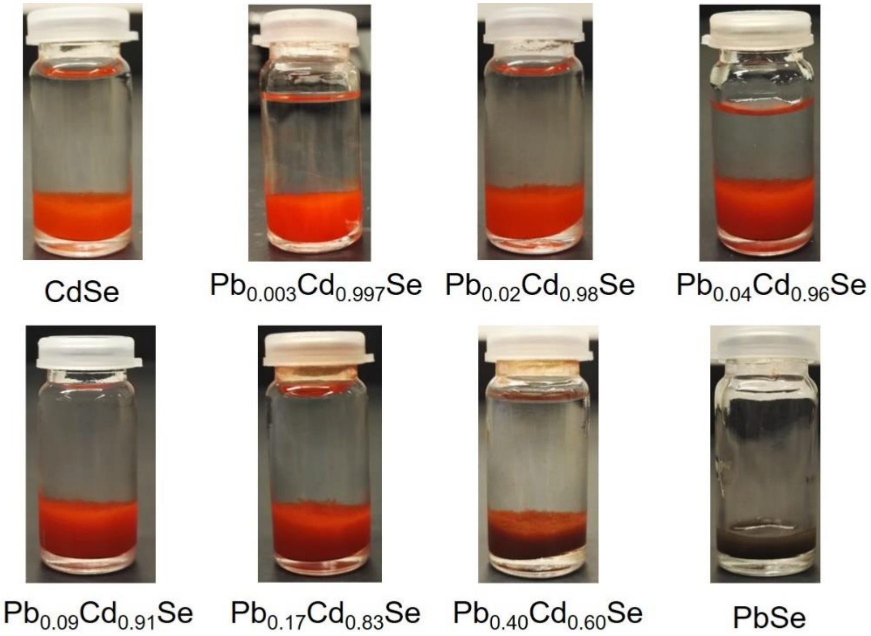

Supplementary Figure 2. Photographs of $Pb_xCd_{1-x}Se$ wet gels (x=0, 0.003, 0.02, 0.04, 0.09, 0.17, 0.40 and 1.0).

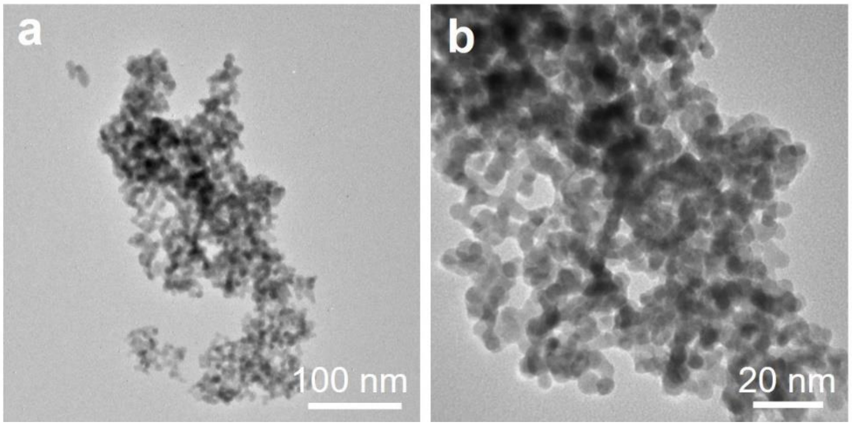

Supplementary Figure 3. a, Low and **b**, high-magnification TEM images of PbSe QD gel.

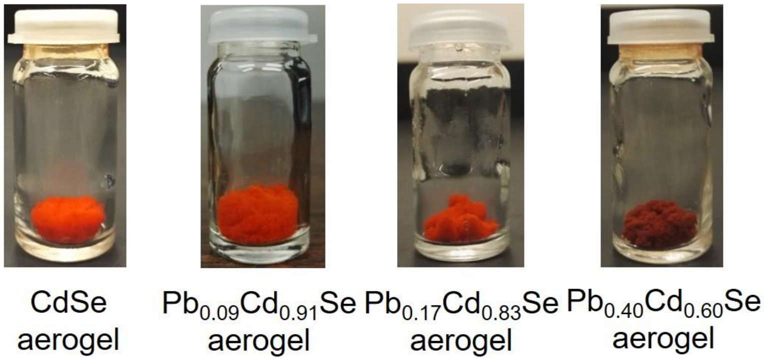

Supplementary Figure 4. Photographs of $Pb_xCd_{1-x}Se$ QD aerogels (x=0, 0.09, 0.17 and 0.40).

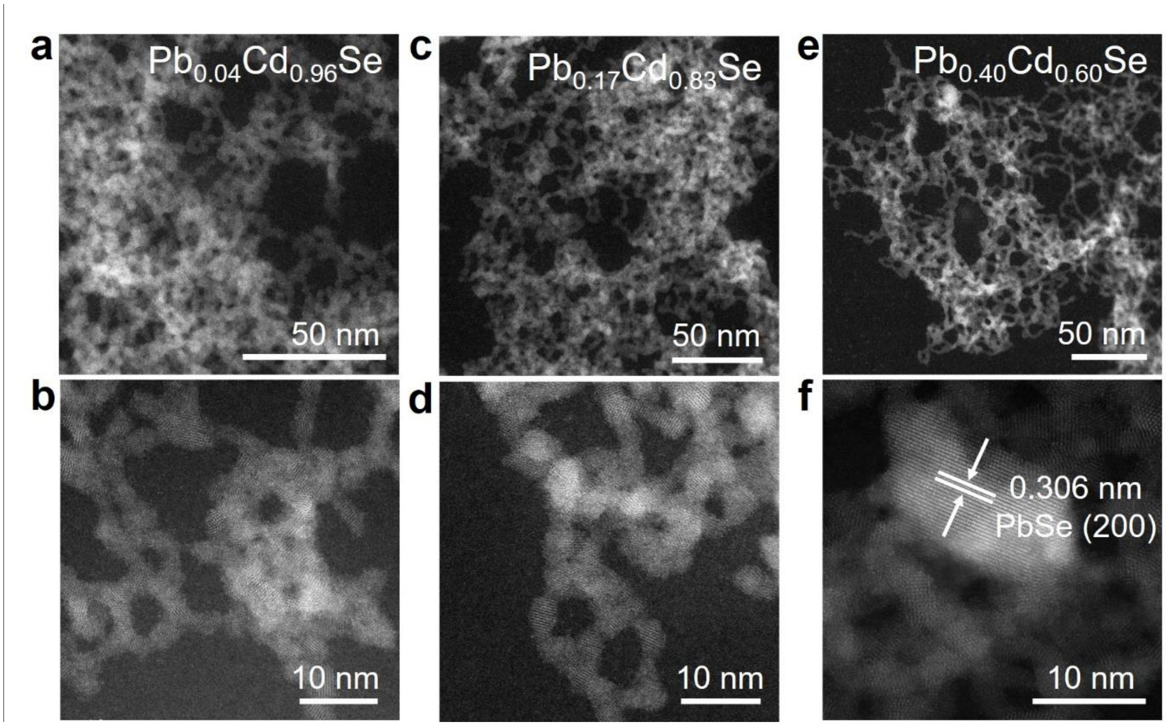

Supplementary Figure 5. **a**, Low and **b**, high-magnification HAADF-STEM images of $Pb_{0.04}Cd_{0.96}Se$ gel. **c**, Low and **d**, high-magnification HAADF-STEM images of $Pb_{0.17}Cd_{0.83}Se$ gel. **e**, Low and **f**, high-magnification HAADF-STEM images of $Pb_{0.40}Cd_{0.60}Se$ gel.

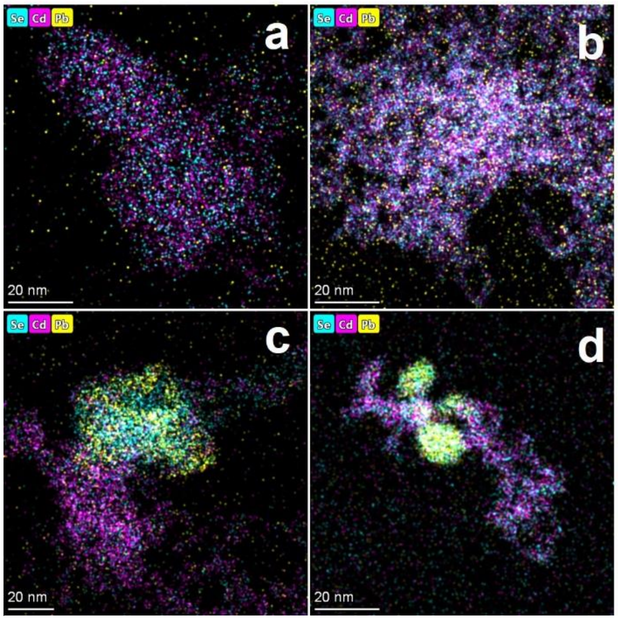

Supplementary Figure 6. Integrated EDS elemental mappings of **a**, $Pb_{0.04}Cd_{0.96}Se$; **b**, $Pb_{0.09}Cd_{0.91}Se$; **c**, $Pb_{0.17}Cd_{0.83}Se$; **d**, $Pb_{0.40}Cd_{0.60}Se$ QD gels.

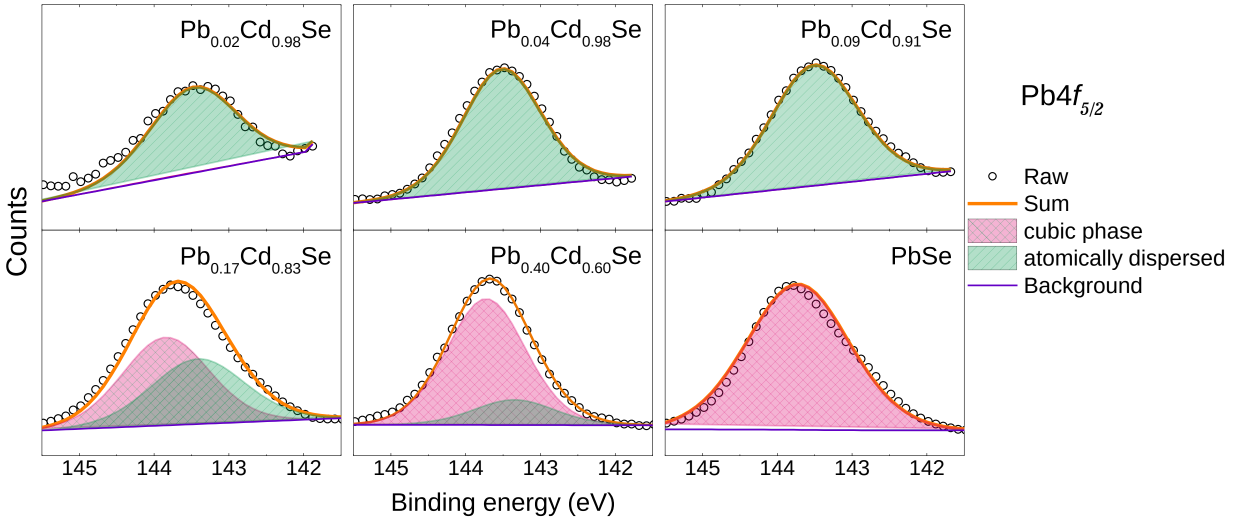

Supplementary Figure 7. XPS peak analysis of Pb4$f_{5/2}$ region for $Pb_xCd_{1-x}Se$ QD gels with x=0.02, 0.04, 0.09, 0.17, 0.40 and 1.0. Fitting was performed using a composite function (30% Lorentzian + 70% Gaussian).

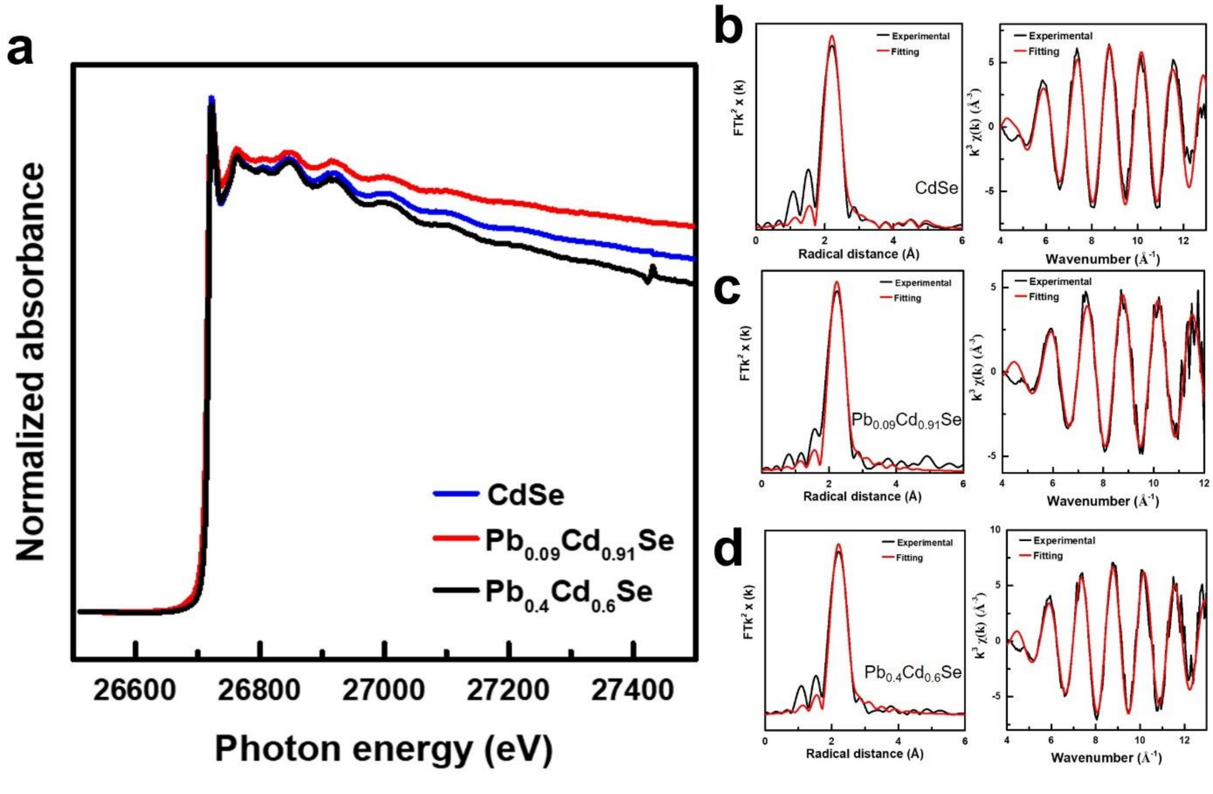

Supplementary Figure 8. a, XANES spectra of CdSe, $Pb_{0.09}Cd_{0.91}Se$, and $Pb_{0.4}Cd_{0.6}Se$ QD gels at Cd K-edge. The EXAFS spectra and fitting results of **b**, CdSe gel; **c**, $Pb_{0.09}Cd_{0.91}Se$ gel; **d**, $Pb_{0.4}Cd_{0.6}Se$ gel.

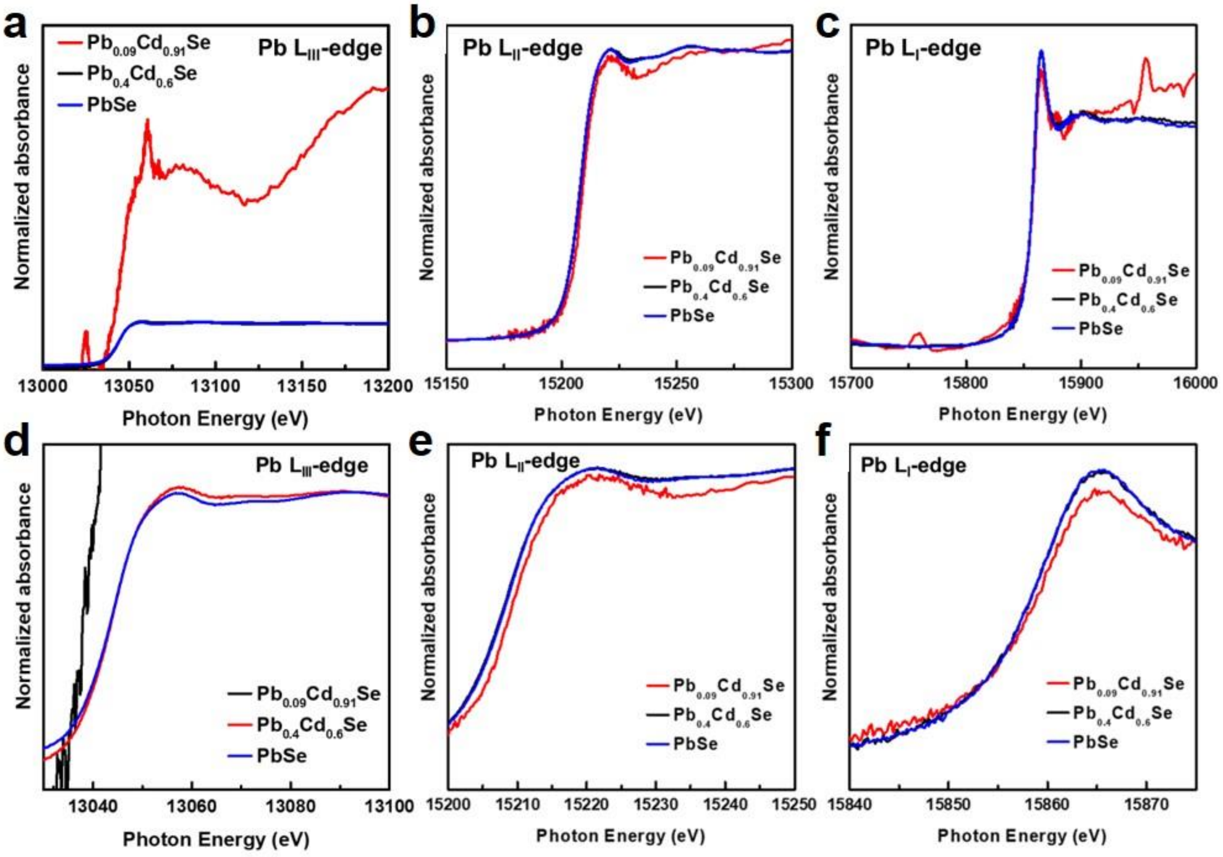

Supplementary Figure 9. XANES spectra of PbSe, $Pb_{0.09}Cd_{0.91}Se$, and $Pb_{0.4}Cd_{0.6}Se$ QD gels at **a**, Pb L_{III} edge; **b**, Pb L_{II} edge and **c**, Pb L_{I} edge. The corresponding expanded views at **d**, Pb L_{III} edge; **e**, Pb L_{II} edge; and **f**, Pb L_{I} edge.

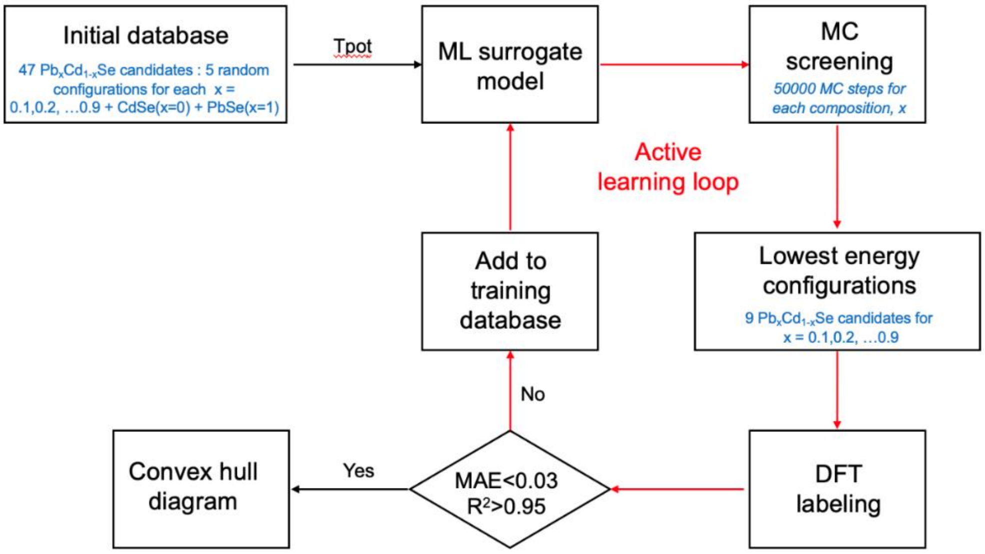

Supplementary Figure 10. Flowchart for the active machine learning process (taking the cubic $Pb_xCd_{1-x}Se$ as an example).

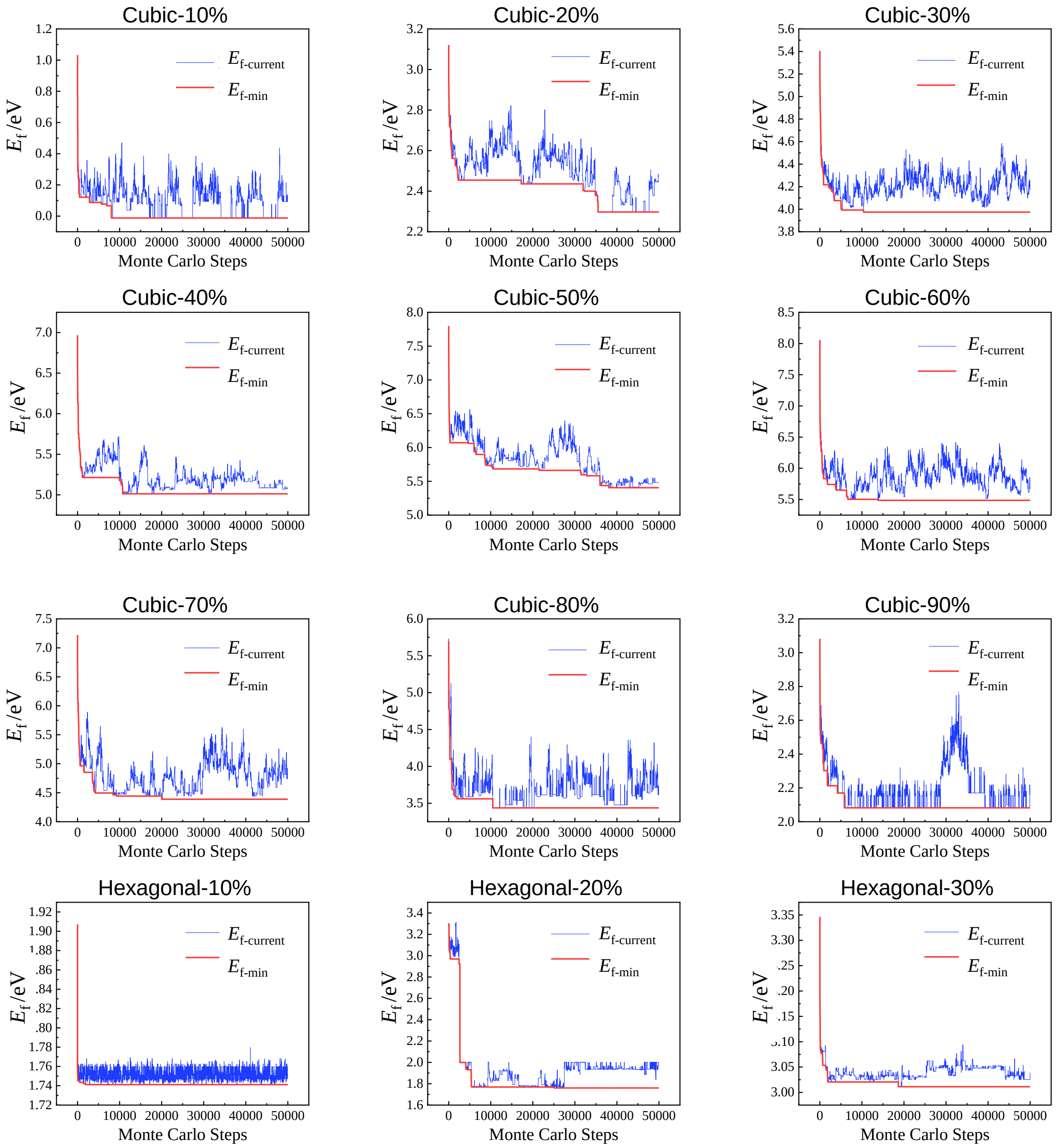

Supplementary Figure 11. The searching process of the most stable geometries at each composition in the last run of the Monte Carlo simulation. Note that the expressions of E_f for cubic and hexagonal phase are different (Cubic : $E_\mathrm{f} = (E_{\mathrm{Pb_xCd_{1-x}Se}} - x \cdot E_{\mathrm{PbSe-cubic}} - (1-x) \cdot E_{\mathrm{CdSe-cubic}})/108$ and Hexagonal : $E_\mathrm{f} = (E_{\mathrm{Pb_xCd_{1-x}Se}} - x \cdot E_{\mathrm{PbSe-hex}} - (1-x) \cdot E_{\mathrm{CdSe-hex}})/108$))

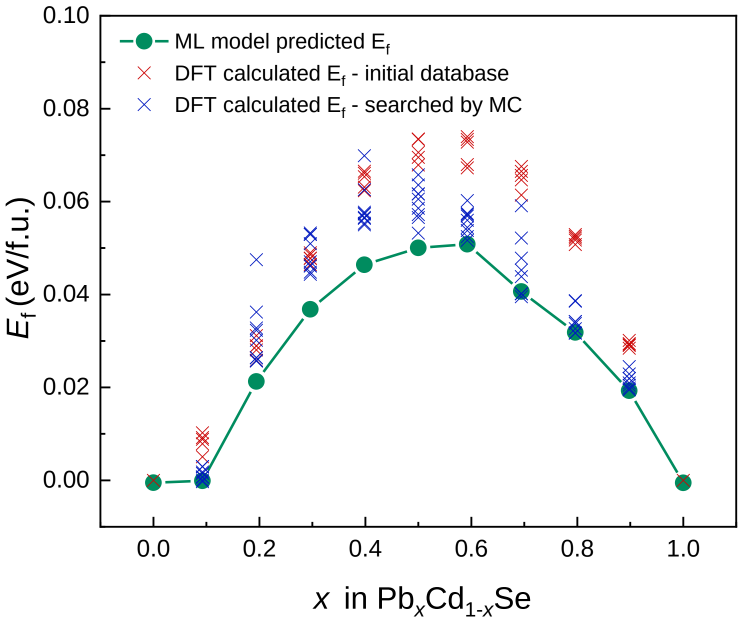

Supplementary Figure 12. The relative formation energy per formular unit for cubic phase. The green points were the E_f predicted by the ML model in last iteration which exhibited high prediction accuracy. It can be concluded that the structures (blue points) searched by MC algorithm were more stable than the structures which were generated randomly (red points), demonstrating the effectiveness of our active learning strategy for searching most stable configurations.

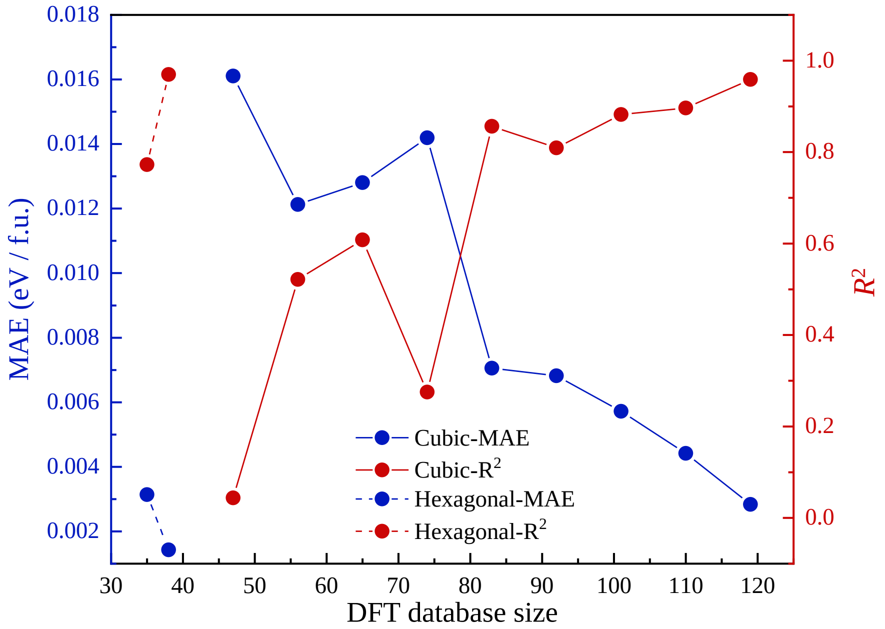

Supplementary Figure 13. The change of MAE and R^2 as a function of the number of active learning loops.

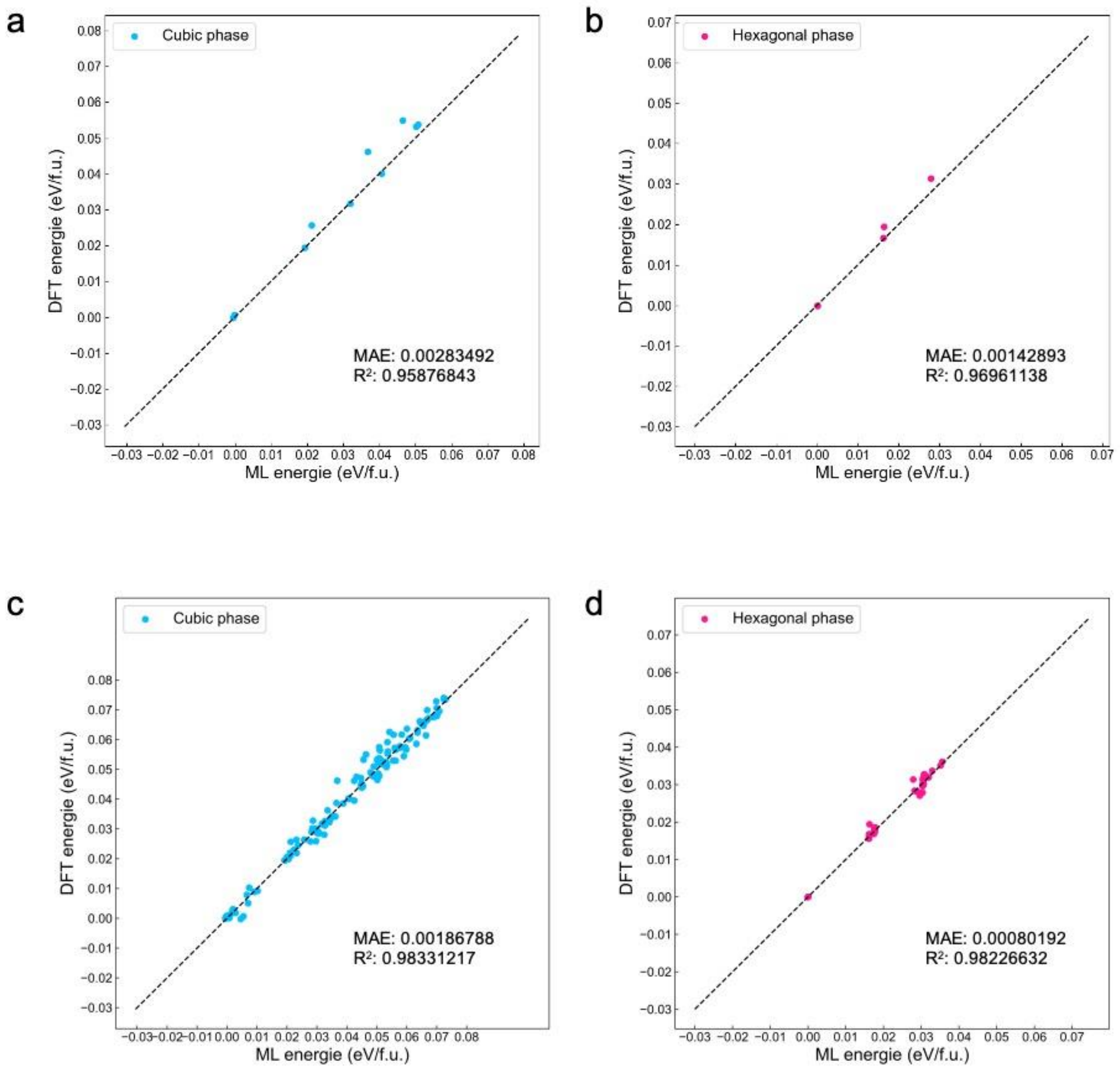

Supplementary Figure 14. The parity plot of cubic and hexagonal phase in the last iteration. **a**, Parity plot of the cubic phase structures searched by the last MC run; **b**, Parity plot of the hexagonal phase structures searched by the last MC run; **c**, Parity plot of all labeled structures in cubic phase; **d**, parity plot of all labeled structures in hexagonal phase.

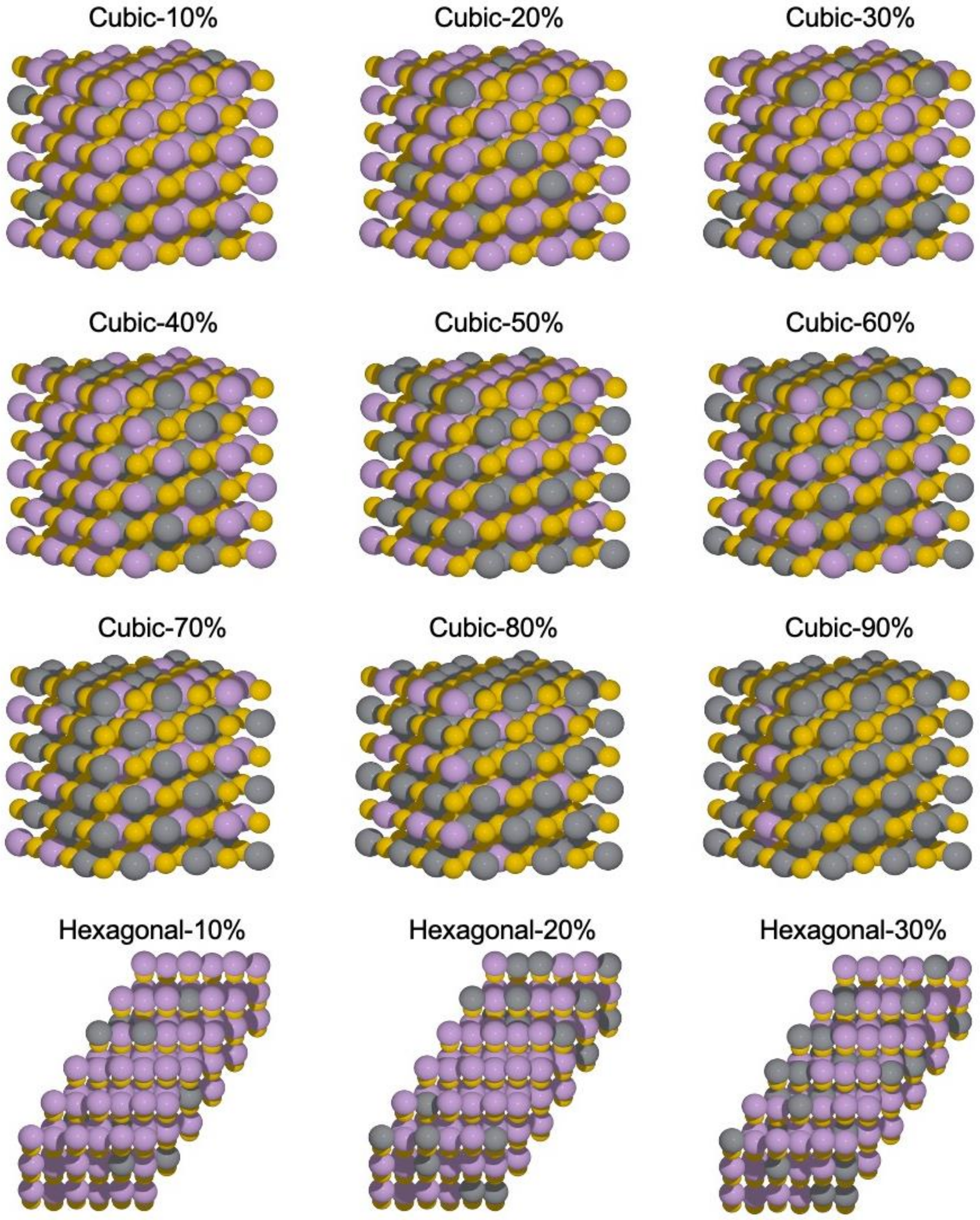

Supplementary Figure 15. The most stable structures for each composition searched by our scheme.

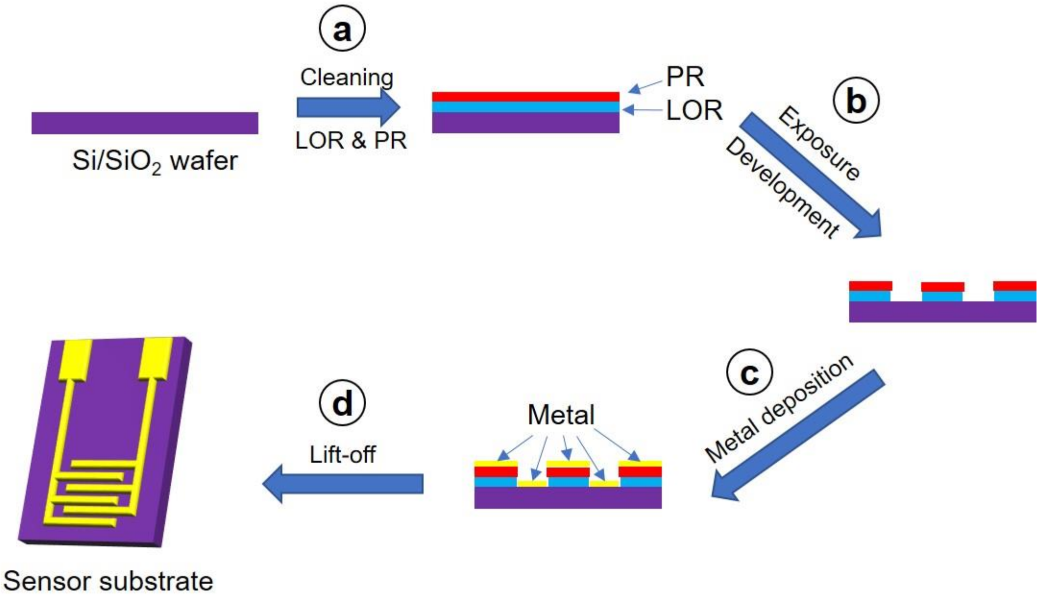

Supplementary Figure 16. Schematic of the sensor substrate fabrication. a, wafer cleaning and LOR/PR spin-coating. b, UV exposure and development of PR. c, metal deposition. d, lift-off. LOR: Photoresist for lift-off; PR: photoresistor.

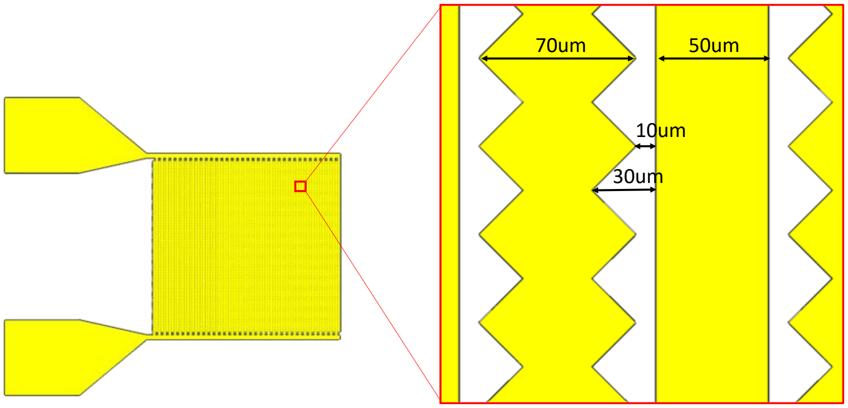

Supplementary Figure 17. Geometry and dimensions of the sensor substrate patterned with Au electrodes.

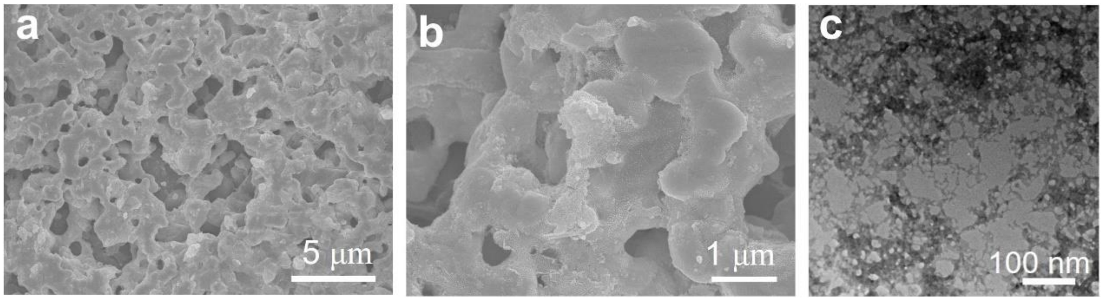

Supplementary Figure 18. a, Low and **b**, high-magnification Field Emission Scanning Electron Microscope (FE-SEM) surface morphology images of $Pb_{0.09}Cd_{0.91}Se$ gel sensor. **c**, TEM image of $Pb_{0.09}Cd_{0.91}Se$ gel sensor film.

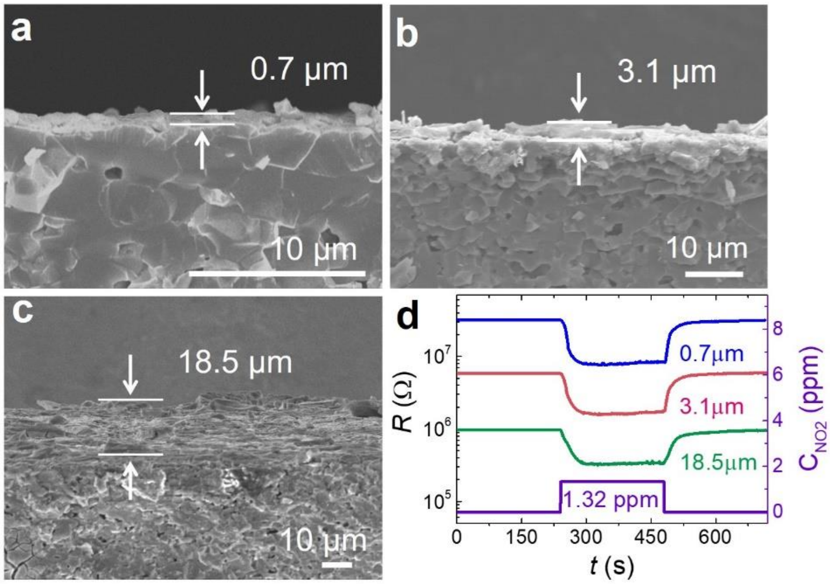

Supplementary Figure 19. FE-SEM cross-sectional images of $Pb_{0.09}Cd_{0.91}Se$ gel sensors prepared by drop-casting **a**, 2 μL; **b**, 10 μL; and **c**, 50 μL wet gel onto the sensor substrate, respectively. **d**, Response-recovery curves of $Pb_{0.09}Cd_{0.91}Se$ gel sensors with a thickness of 0.7 μm, 3.1 μm, and 18.5 μm at 1.32 ppm NO_2 at room temperature.

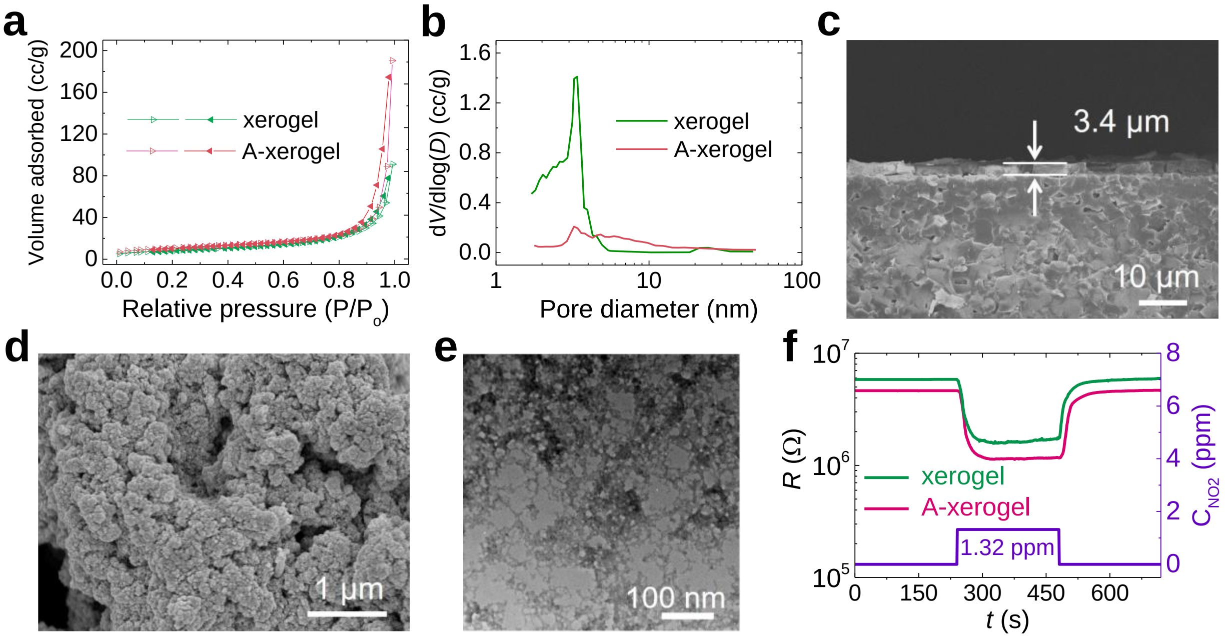

Supplementary Figure 20. **a**, Nitrogen adsorption-desorption isotherms; **b**, Barrett–Joyner–Halenda pore size distributions of $Pb_{0.09}Cd_{0.91}Se$ xerogel and A-xerogel. **c**, Cross-section FE-SEM image of $Pb_{0.09}Cd_{0.91}Se$ A-xerogel sensor; **d**, FE-SEM and **e**, TEM image of the $Pb_{0.09}Cd_{0.91}Se$ A-xerogel sensor film; **f**, Response-recovery curve of $Pb_{0.09}Cd_{0.91}Se$ xerogel and A-xerogel sensors in response to 1.32 ppm NO_2 at room temperature.

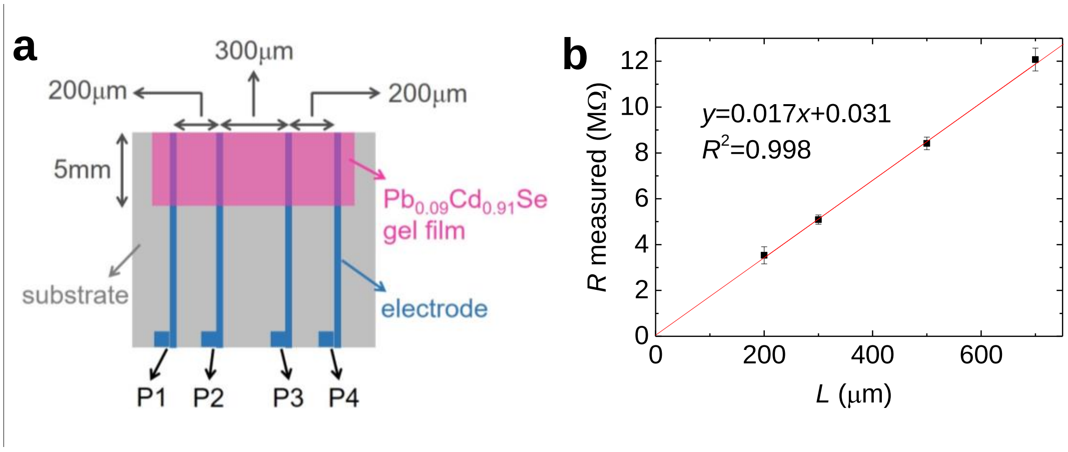

Supplementary Figure 21. **a**, Schematic diagram of the sensor substrate design for measuring contact resistance for the $Pb_{0.09}Cd_{0.91}Se$ gel. **b**, Extrapolated line fitting for determining the contact resistance from the plot of total resistance ($R_{measured}$) versus the distance between two electrodes (L) for each unique combination (e.g., P1-P2, P1-P3, etc.) according to $R_{measured} = 2R_{contact} + R_{gel} = 2R_{contact} + \rho L/A$, where ρ is the gel resistivity and A is the cross-sectional area. The y-axis intercept (0.031 MΩ) is twice the contact resistance. The error bars are the standard deviations from three independent measurements.

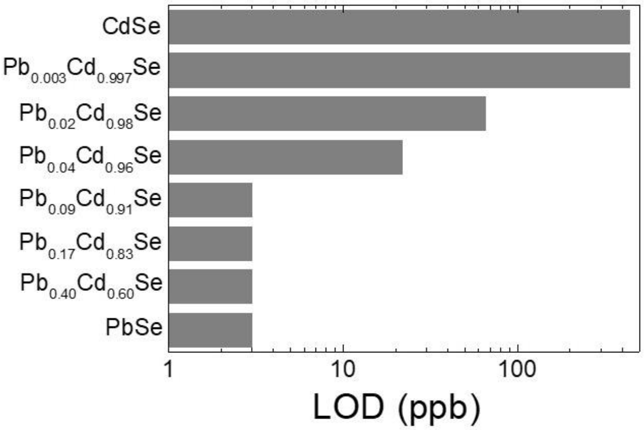

Supplementary Figure 22. Limit of detection (LOD) of $Pb_xCd_{1-x}Se$ gel sensors (x=0, 0.003, 0.02, 0.04, 0.09, 0.17, 0.40 and 1.0).

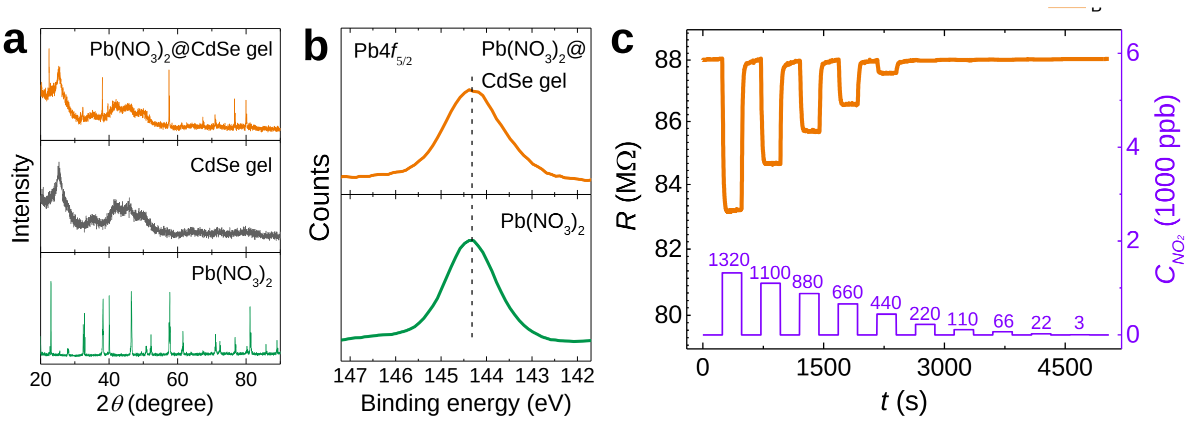

Supplementary Figure 23. a, XRD patterns of $Pb(NO_3)_2$@CdSe gel, CdSe QD gel, and $Pb(NO_3)_2$. **b**, XPS results of the Pb4$f_{5/2}$ region for $Pb(NO_3)_2$@CdSe gel and $Pb(NO_3)_2$. **c**, Response–recovery curve of a $Pb(NO_3)_2$@CdSe gel sensor in response to NO_2 (3 ppb–1.32 ppm) at room temperature. $Pb(NO_3)_2$@CdSe gel is a CdSe gel physically loaded with $Pb(NO_3)_2$ with a Pb:Cd ratio of 0.09:0.91, prepared by mixing 0.005 mmol $Pb(NO_3)_2$ with CdSe xerogel ($n_{[Cd2+]}$=0.05 mmol) in a nonpolar solvent, hexane, to prevent cation exchange between Pb salt and CdSe QD gel.

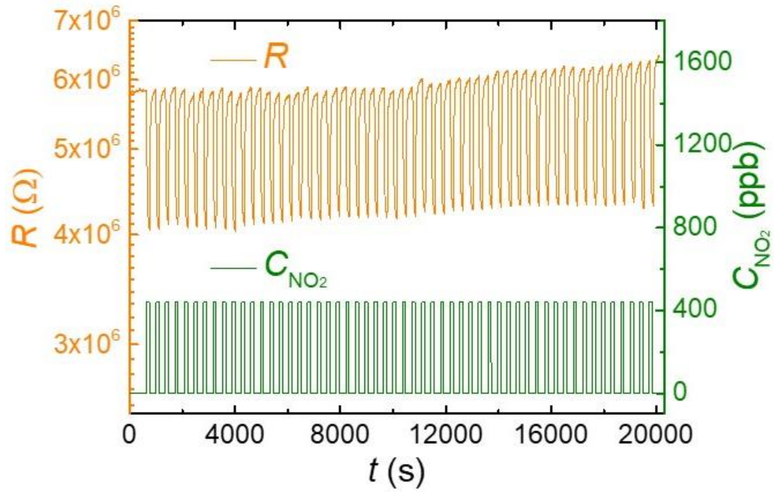

Supplementary Figure 24. A typical electrical resistance-time trace for a $Pb_{0.09}Cd_{0.91}Se$ gel sensor during the response-recovery test.

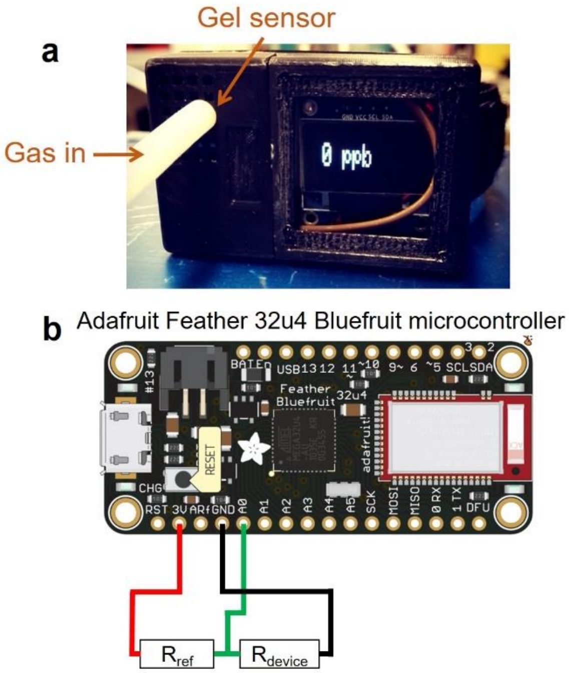

Supplementary Figure 25. a, Photograph of the home-made wireless portable device for NO_2 gas sensing. **b**, Schematic diagram of device design. R_{ref} is connected to the supply pin (3.3 V) and to the analog input pin A0 of an Adafruit Feather 32u4 BlueFruit microcontroller. The sensor (R_{device}) is connected to A0 and to the ground pin (GND) of the microcontroller. This illustration is a derivative of the work by Alberto Piganti available at https://pin.it/1eZUCIG used under a CC-BY-SA license (creativecommons.org/licenses/by-sa/4.0/)

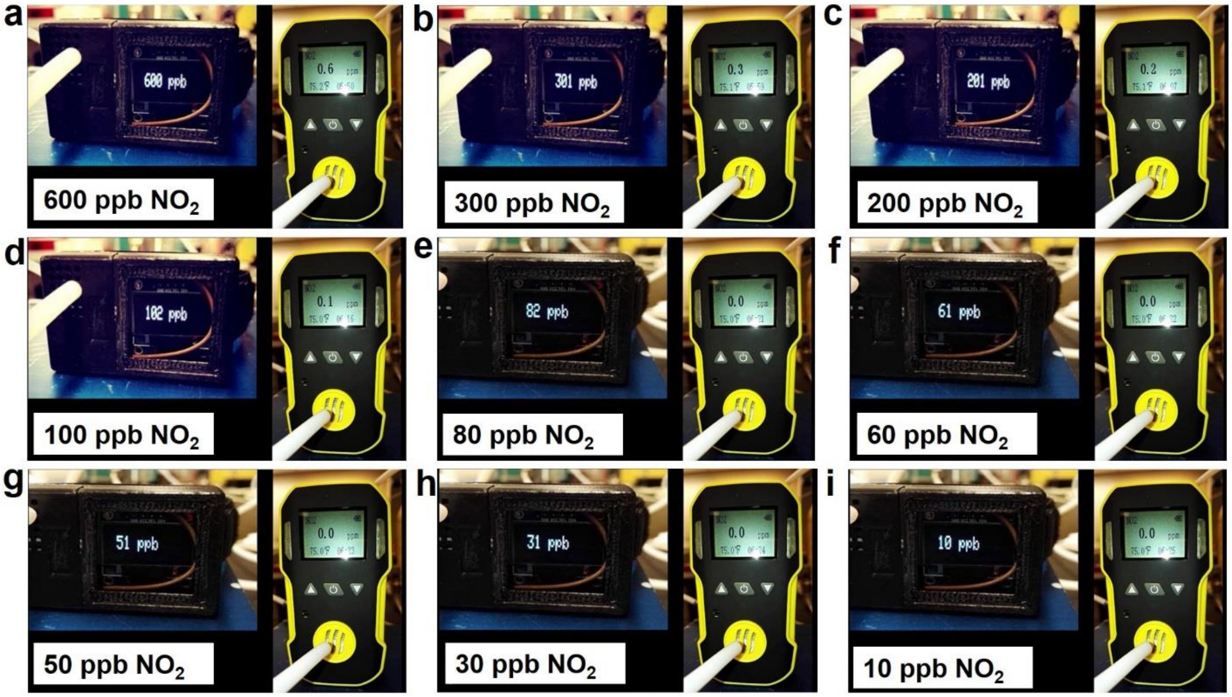

Supplementary Figure 26. Photographs of the readouts of a $Pb_{0.09}Cd_{0.91}Se$ gel portable device and a commercial NO_2 device purchased from amazon (Purchase link: https://www.amazon.com/FORENSICS-Anti-slip-Explosion-Adjustable-Vibration/dp/B07BDNYR86) at different NO_2 concentrations of **a**, 600 ppb; **b**, 300 ppb; **c**, 200 ppb; **d**, 100 ppb; **e**, 80 ppb; **f**, 60 ppb; **g**, 50 ppb; **h**, 30 ppb; and **i**, 10 ppb.

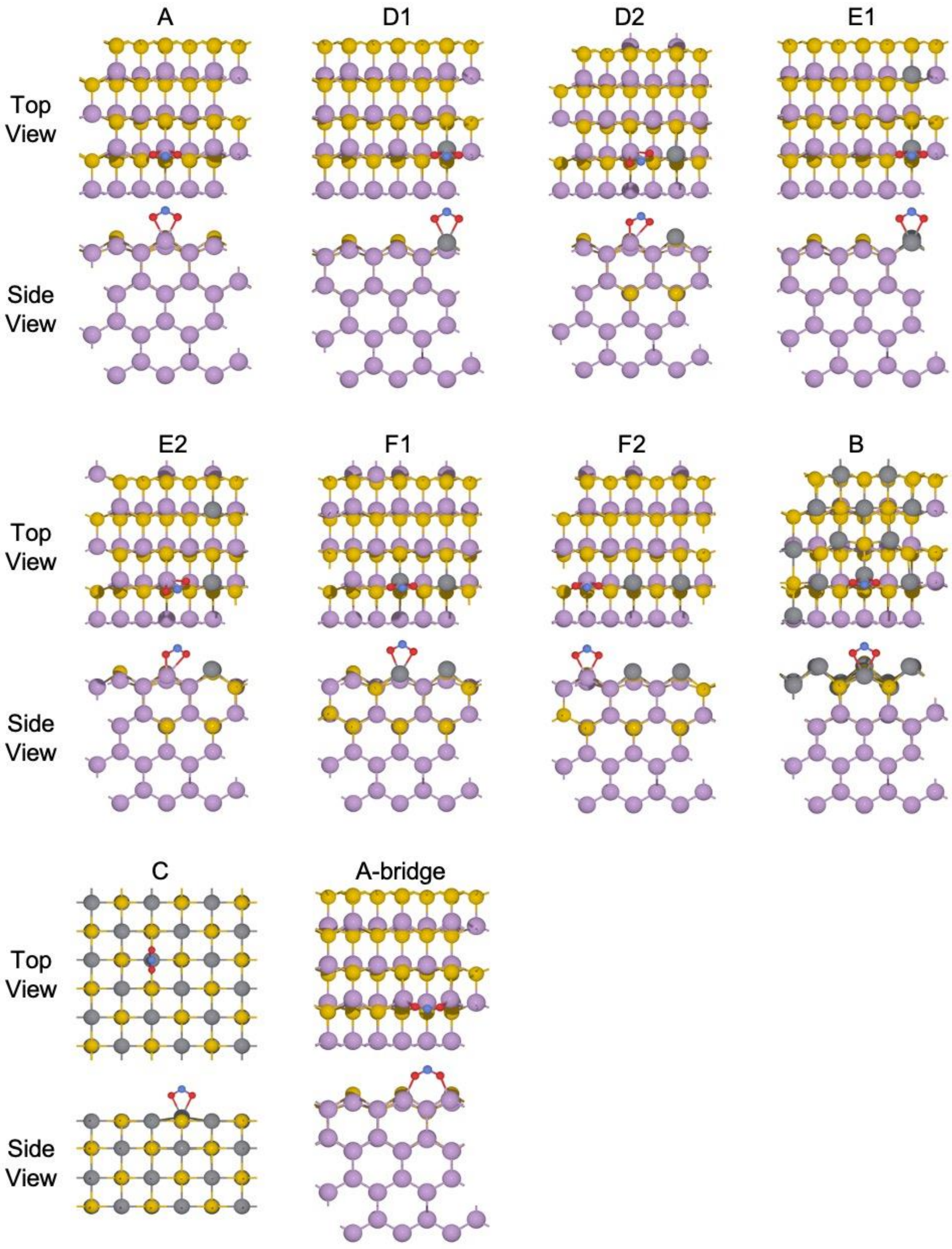

Supplementary Figure 27. The optimal adsorption geometries (top and side view) for NO_2 adsorption on various surface structures. It worth noting that for the pristine hexagonal CdSe (100) surface, the adsorption energy of the bridge adsorption geometry (A-bridge) was slightly stronger (~ 0.04 eV) than that of the chelating adsorption geometry (A). However, the charge transfer was much smaller (0.05 e). Thus, only the chelating adsorption geometry was discussed in the main text. For all other type of surfaces and binding sites, chelating is the most stable geometry for NO_2 adsorption.

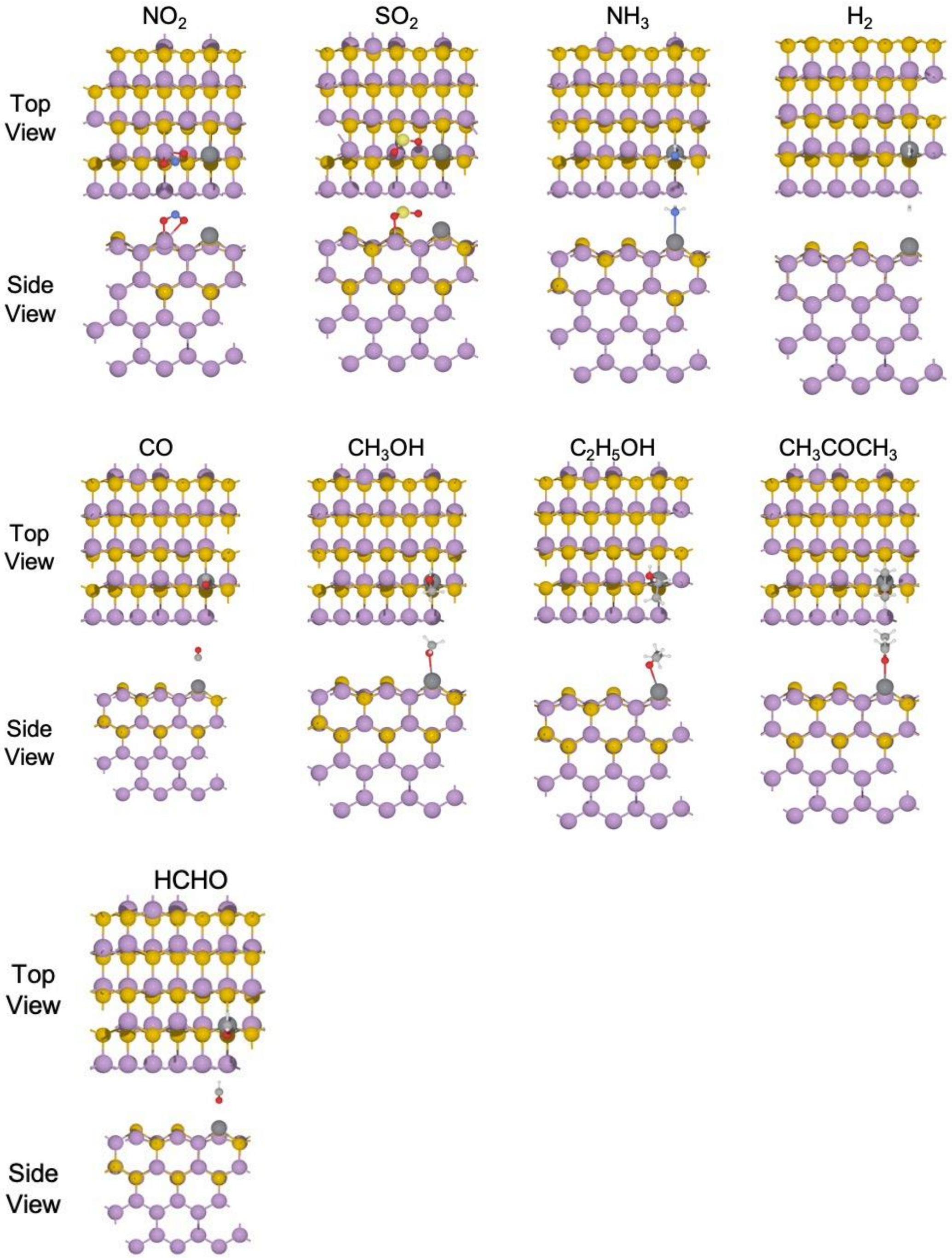

Supplementary Figure 28. The most stable adsorption geometries (top and side view) for various interfering gases adsorbed on D2.
