# Supplementary material for: Atomically dispersed Pb ionic sites in PbCdSe quantum dot gels enhance room-temperature NO2 sensing
Source: Nat Commun. 2021 Aug 12;12:4895. doi: 10.1038/s41467-021-25192-4 (PMC8361172; doi:10.1038/s41467-021-25192-4)
Supplement: Supplementary file 3 — Description of Additional Supplementary Files [file 41467_2021_25192_MOESM3_ESM.pdf]

Description of Additional Supplementary Files

Supplementary Movie 1: The video of the readouts of a $Pb_{0.09}Cd_{0.91}Se$ gel portable device and a commercial NO_2 device at different NO_2 concentrations (10-600 ppb).
